# Genomic Insights into *Pasteurella multocida* Serotype B:2 from Hemorrhagic Septicemia Outbreaks in Wildlife and Livestock in Kazakhstan

**DOI:** 10.3390/pathogens14121273

**Published:** 2025-12-11

**Authors:** Asylulan Amirgazin, Gulzhan Yessembekova, Assel Akhmetova, Talgat Karibayev, Kassym Mukanov, Elena Shevtsova, Bolat Abdigulov, Sarsenbay Abdrakhmanov

**Affiliations:** 1 National Center for Biotechnology, Astana 010000, Kazakhstan; 2 Department of Veterinary Sanitation, Faculty of Veterinary and Animal Husbandry Technology, S. Seifullin Kazakh Agrotechnical Research University, Astana 010011, Kazakhstan; 3 National Reference Veterinary Center, Astana 010000, Kazakhstan

**Keywords:** *Pasteurella multocida*, serogroup B, serotype B:2, *Saiga tatarica*, hemorrhagic septicemia

## Abstract

Outbreaks of hemorrhagic septicemia (HS) caused by *Pasteurella multocida* serogroup B are endemic in Kazakhstan. These outbreaks have repeatedly led to mass mortality events among wild saigas and economic losses to farms. The aim of this study was to conduct the first whole-genome sequencing (WGS) and analysis of *P. multocida* genomes associated with HS cases in saigas and livestock in Kazakhstan. In this study, WGS was performed on 22 *P. multocida* isolates obtained from saigas and livestock. A comparative genomic analysis of *P. multocida* isolates from Kazakhstan and publicly available genomes was performed. All isolates belonged to the B:2:ST122 genotype and formed distinct phylogenetic clusters based on outbreaks in saiga populations and livestock. Clustering also corresponded to identified mutations in virulence genes. Isolates recovered from the 2015 mass mortality of saigas in the Betpak-Dala population were found to have a deletion of the flp1 gene. This observation emphasizes the study of the role of Flp pili in HS pathogenesis. Comparison of the *P. multocida* B:L2:ST122 genomes revealed low virulence gene diversity and an open pangenome. Prophage annotation did not identify virulence or pathogenicity genes. The obtained results will be useful for future studies of HS pathogenesis.

## 1. Introduction

*Pasteurella multocida* is an opportunistic bacterium capable of causing both endemic and sporadic outbreaks of epizootic pasteurellosis in a wide range of domestic and wild mammals and birds [1,2]. The majority of infection cases were recorded in cattle, birds, pigs, rodents, domestic cats and dogs, as well as in humans [3]. *Pasteurella multocida* causes a variety of clinical syndromes in wild and farm animals, including fowl cholera, progressive atrophic rhinitis and pneumonia in pigs and rabbits, lower respiratory tract infections and hemorrhagic septicemia [4].

Hemorrhagic septicemia (HS) is an acute septicemic disease that predominantly affects ungulates [5,6]. It is characterized by rapid disease progression (from several hours to several days) and high mortality levels, reaching up to 100% within affected herds [7,8,9]. The causative agent, *P. multocida* produces a capsule belonging to serogroup B or E (prevalent in Asia or Africa, respectively) and is classified into antigenically different lipopolysaccharide serovars 2 or 5 [10].

Modern multiplex PCR assays used to classify *P. multocida* into subspecies (*multocida*, *septica*, *gallicida*) [11], to differentiate into capsular (A, B, D, E and F) [12] and to identify lipopolysaccharide (LPS: L1-L8) types [13] have replaced traditional biochemical and serological testing methods [14,15,16]. Epidemiological analyses are mainly based on genotyping and comparison of isolates using two classical MLST schemes called RIRDC [17] and Multi-host [18] (hereinafter ^RIRDC^MLST and ^mh^MLST), which are available in PubMLST [19]. Genotypes of the “Capsule:LPS:MLST” type show limited correlation with the pasteurellosis syndromes they cause and their host animals [3]. For example, HS of ungulates is associated with genotype B:L2:^RIRDC^ST122 [20].

HS was proven to be the cause of mass mortality of saiga antelopes (*Saiga tatarica tatarica*) in 2015 [21]. Similar die-offs were periodically recorded in the Betpak-dala population (1981—15%, 1988—73%, 2015—88%), the Ural population (1984—73%, 2010—75%) and the Ustyurt population (2015—10,358 animals) [22]. Previous studies during historically similar outbreaks of pasteurellosis in saiga in the same areas suggested that unusually high humidity and temperature during the calving period may have triggered disease development [22]. However, an alternative hypothesis suggested a genetic basis for high virulence or pathogenicity of the pathogen [21]. *P. multocida* isolates recovered from saiga carcasses died in the 2010–2015 mass mortality events were identified as genotype B:^mh^ST64 (^RIRDC^ST122) with 15 virulence genes detected (*ptfA*, *ompA*, *ompH*, *oma87*, *plpB*, *fimA*, *hsf-2*, *pfhA*, *exbB*, *tonB*, *hgbA*, *fur*, *nanB*, *nanH* and *pmHAS*) [23]. To date, no whole-genome sequencing and comparative genomic analysis of *P. multocida* isolates associated with saiga mass die-off events in Kazakhstan was conducted.

Saiga antelopes were previously shown as a possible source of infection for farm animals [24]. Due to free-range pasture farming in Kazakhstan, wildlife and livestock often share the same grazing areas. It is common that infectious diseases are transmitted within livestock and between farm animals and wildlife. Thus, saiga antelopes with pasteurellosis can serve as a source of the pasteurellosis pathogen for farm animals, including cattle [23]. Studying factors driving pasteurellosis transmission patterns within and between livestock and wildlife is essential for improving disease control. Current study includes samples collected from poultry and livestock infected with pasteurellosis, as well as from saiga collected during outbreaks. This study is the first such analysis based on archived isolates collected from saiga and livestock in Kazakhstan.

## 2. Materials and Methods

### 2.1. Isolates Collection

Pathological specimens from wildlife and livestock animals with HS were sent to the National Reference Center for Veterinary (NRCV) of the Committee of Veterinary Control and Supervision of the Ministry of Agriculture of the Republic of Kazakhstan during outbreak investigation. All isolates were recovered from the parenchymatous organs (liver, kidneys, lungs) of deceased animals, including saiga, cattle, horse and poultry.

Homogenized pathological material was prepared as a 10% suspension in sterile saline and used to isolate *Pasteurella multocida* and test pathogenicity by infecting laboratory mice (see Section 2.2). The suspension was used for culturing on meat-peptone agar (HiMedia, Mumbai, India) for 24 h at 37 °C. Individual colonies were then selected for bacterial growth in meat-peptone broth (HiMedia, India) for 24 h at 37 °C.

Genomic DNA was isolated from bacteriological cultures using the QIAamp DNA Mini Kit (Cat. no. 51306, QIAGEN, Hilden, Germany). Species identification of samples was performed by sequencing the *16S rRNA* gene on a 3730xl DNA Analyzer sequencer (Applied Biosystems, Carlsbad, CA, USA) using the BigDye Terminator v3.1 Cycle Sequencing Kit (Applied Biosystems), according to the manufacturer’s instructions. Amplification of the *16S rRNA* gene was performed using the universal primers 8F (5′-AGAGTTTGATCCTGGCTCAG-3′) and 806R (5′-GGACTACCAGGGTATCTAAT-3′) [25]. Species identification of the obtained sequences was carried out using Nucleotide BLAST internet resource (https://blast.ncbi.nlm.nih.gov, accessed on 1 June 2025) based on the “16S ribosomal RNA sequences” database.

As a result, gDNA samples were obtained for 22 *Pasteurella multocida* isolates from saiga (*n* = 15), cattle (*n* = 4), horses (*n* = 2), and poultry (*n* = 1). Key metadata for the isolates studied are presented in Table 1.

### 2.2. Testing the Pathogenicity of Isolates

All work involving laboratory animals was carried out at the National Reference Center for Veterinary, the only reference laboratory in Kazakhstan authorized to monitor infectious diseases in wildlife. The center has a BSL-3 facility where experiments involving laboratory mice were conducted. All bacteriological procedures, including pathogenicity testing of isolates, were performed in accordance with the National Standard of the Republic of Kazakhstan ST RK 3508 “Methods of Laboratory Diagnostics of Pasteurellosis”.

To test the pathogenicity of each isolate, experiments were conducted involving two groups of BALB/c mice. The criteria for inclusion of mice in the pathogenicity testing of isolates were: physical health, male sex and age in the range from 6 to 20 weeks. To ensure statistical reliability, reproducibility, and the use of a minimal number of laboratory animals, each group (test and control) included at least three mice. Mice in the test group were infected with 0.1 mL of a 10% suspension of the pathological material. Mice in the control group were injected with 0.1 mL of sterile physiological saline solution. There were no randomization or blinding used in this analysis. To minimize potential confounders, each mouse was kept in a separate cage. A total of 132 mice were used to test the pathogenicity of the 22 *P. multocida* isolates.

Cardiac blood from deceased mice was cultured using meat-peptone agar (HiMedia, India) for 24 h at 37 °C. Single colonies were then selected and grown in meat-peptone broth (HiMedia, India) for 24 h at 37 °C. Genomic DNA isolation and species identification were conducted as described in Section 2.1. *P. multocida* isolates were considered pathogenic if they caused death of mice within 24 to 36 h after infection. Since the results were homogeneous binary indicators, statistical methods were not used.

### 2.3. Whole-Genome Sequencing and Genome Assembly

DNA libraries were prepared using the Nextera XT DNA Library Preparation Kit (Cat. no. FC-131-1096, Illumina, San Diego, CA, USA) according to the manufacturer’s instructions. Sequencing was performed on an Illumina MiSeq sequencer using the Illumina MiSeq Reagent Kit v3 (600-cycle, Cat. no. MS-102-3003, Illumina). Raw read quality control was performed using FastQC [26]. Raw data trimming was performed using Seqtk v1.3 (https://github.com/lh3/seqtk, accessed on 1 June 2025) and Sickle v1.33 (https://github.com/najoshi/sickle, accessed on 1 June 2025). Genome assemblies were performed using SPAdes v3.15.5 [27] and Skesa v2.5.1 [28]. De novo assembly metrics were assessed using QUAST v5.3.0 [29]. Assembly completeness and contamination were assessed using CheckM v1.2.3 [30] using the “lineage_wf” pipeline and “f_Pasteurellaceae (UID4933)” markers. Sequencing depth was calculated by aligning trimmed reads to the corresponding assemblies using BWA v0.7.19 [31], determining the alignment depth using SAMtools v1.13 [32], and calculating the median value using Python3 (https://www.python.org, accessed on 1 June 2025). All parameters of the software tools mentioned in this study are described in Supplementary Table S1.

### 2.4. Phylogenetic Analysis

To identify phylogenetically related strains to the isolates studied in current analysis, 1893 *P. multocida* genome sequences were imported from the NCBI database (https://www.ncbi.nlm.nih.gov/datasets/genome/?taxon=747, accessed on 1 June 2025) (import date: June 2025). The “EXCLUDE: Atypical genomes, Metagenome-assembled genomes (MAGs)” filters were used for import. Downloaded and studied genomes were analyzed using Snippy v4.6.0 (https://github.com/tseemann/snippy, accessed on 1 June 2025) with default settings and the *P. multocida ATCC_43137* (CP008918.1) genome as a reference sequence. The resulting “core.full.aln” file was used to construct a phylogenetic tree based on the neighbor-joining algorithm using RapidNJ v2.3.3 (https://github.com/somme89/rapidNJ, accessed on 1 June 2025). Phylogenetic tree was visualized using the iTOL online resource (https://itol.embl.de, accessed on 1 June 2025) [33]. Strains that clustered close to studied isolates were selected for subsequent phylogenetic analysis.

A cgSNP-based phylogenetic tree was constructed using the Maximum parsimony (MP) algorithm to estimate phylogenetic relationships and genetic distances between samples. Genome assemblies of the studied isolates and the selected genomes of phylogenetically related strains were re-analyzed using Snippy to determine cgSNPs. The resulting “core.full.aln” file was processed by Gubbins v3.4.1 [34] to remove regions with a high density of recombination events. A maximum-parsimony tree was constructed based on the cgSNPs using the “dist.hamming” function with default parameters from the Phangorn v2.12.1.3 package [35]. For visualization of phylogenetic trees GrapeTree v1.5.0 [36] was used with subsequent additions in the Inkscape v1.4.2 graphics editor (https://inkscape.org/, accessed on 1 June 2025).

### 2.5. Genotyping

ANIclustermap v1.4 software tool was used to identify *septica* and *multocida* subspecies [37] with the addition of the reference genomes *P. multocida* subsp. *septica NCTC11995* (GCF_900454845.1) and *P. multocida* subsp. *multocida NCTC10322* (GCF_900187275.1) to the analysis. *Gallicida* subspecies were identified by searching for the *gatD* gene (WP_005753626) using the BLAST+ v2.17.0 package [38]. Genotyping using the RIRDC MLST and Multi-host MLST panels was performed on the PubMLST online resource (https://pubmlst.org, accessed on 1 June 2025). Nucleotide sequences corresponding to capsule and LPS biosynthesis genes, as well as virulence genes, were detected using fromAssembly2Feature software (https://github.com/LPerlaza/fromAssembly2Feature, accessed on 1 June 2025) based on PastyVRDB database [39] (Supplementary File S1). Confirmation of gene deletions or mutations, as well as obtaining gene sequences missing from the genome assemblies, was performed by mapping raw reads to reference gene sequences using an in-house script available in Supplementary File S2. Visualization of the fromAssembly2Feature results (percentage of amino acid identity) was performed using the Pheatmap v1.0.13 package [40]. Comparison of amino acid sequences and visualization of tad loci were performed by local alignment using BLAST+ v2.17.0 and Geneviewer v0.1.11 (https://nvelden.github.io/geneviewer/, accessed on 1 June 2025). Integrative and conjugative elements (ICEs) were detected using the ICEfinder 2.0 software [41]. Plasmid sequences were identified using PlasmidHunter v1.4 (https://github.com/tianrenmaogithub/PlasmidHunter, accessed on 1 June 2025) with further comparison of the results in Nucleotide BLAST (https://blast.ncbi.nlm.nih.gov, accessed on 1 June 2025) using the “core_nt” database. Antimicrobial resistance genes (AMR) or resistance-associated mutations in *P. multocida* genomes were assessed using AMRFinderPlus v4.0.23 [42] and the ABRicate v1.0.0 tool (https://github.com/tseemann/abricate, accessed on 1 June 2025) with the ResFinder database.

### 2.6. Prophage Analysis

Prophage sequences were searched using the PHASTEST online resource (https://phastest.ca, accessed on 1 June 2025) [43]. Prophage sequence clustering was also performed using ANIclustermap v1.4. Prophage genome annotation and visualization were performed using Pharokka v1.8.0 [44], Phold v1.0.0 [45] and LoVis4u v0.1.2 [46].

### 2.7. Pangenome Analysis

Genome annotation was performed using Prokka v1.14.5 [47]. Protein amino acid sequences of 1893 *P. multocida* genomes were imported from NCBI using the same workflow described above. Sequences were then de-duplicated using SeqKit v2.10.1 [48] and used as a database for annotation using the “--proteins” parameter. Pangenome analysis was performed using Panaroo v1.5.2 software [49] and the additional script “panaroo-filter-pa”. The resulting file “gene_presence_absence_filt_pseudo_length_frag” was analyzed using an in-house script, the code for which is available in Supplementary File S3. Functional annotation of representative sequences of orthologous genes (COGs) clusters was performed using eggNOG-mapper v2.1 online resource [50] with default parameters. Functional annotation visualization was performed using Matplotlib v3.10.7 (https://pypi.org/project/matplotlib/, accessed on 1 June 2025). Analysis and visualization of pangenome curves were performed using the R package Pagoo v0.3.17 [51]. Gene association analysis was performed using Scoary2 v0.0.15 [52].

### 2.8. Ethical Approval

Confirmation of the pathogenicity of archived isolates of *P. multocida* was carried out by infecting white laboratory mice in accordance with the National Standard of the Republic of Kazakhstan ST RK 3508 “Methods of laboratory diagnostics of pasteurellosis”, which includes bioethical approval.

Ethical approval for current research study was received from an ethical committee of the LLP National Center for Biotechnology (Protocol No. 6 dated 5 October 2022).

Generative artificial intelligence (GenAI) was not used in this work.

## 3. Results

### 3.1. Sampling and Isolates Characterization

Collected parenchymatous organs of saiga, cattle, and horses showed typical clinical signs of HS. The exception was one saiga carcass corresponding to *LPG-26* isolate, which showed lesions only in the lungs. All tested isolates caused death of laboratory mice within 10 h of subcutaneous administration. No clinical signs of the disease were observed in the control groups.

### 3.2. Genome Assembly Evaluation

As shown in Table 2, the number of assembled contigs ranged from 20 to 94, with median sequencing depths ranging from 40× to 97×. Completeness values for the resulting genomes ranged from 98.41% to 99.55%. Contamination values ranged from 0.00% to 0.11%. GC% values ranged from 40.16% to 40.35%. The *N50* metric ranged from 64,640 to 537,706. Genome assembly sizes ranged from 2,264,621 bp to 2,496,223 bp. Based on the average nucleotide identity of the genomes and the absence of the *gatD* gene in the assemblies, all isolates were classified as belonging to the subspecies *multocida*.

### 3.3. Genotypes of the Studied Isolates

Corresponding genotypes of the “Capsule:LPS:MLST” type were determined for all isolates (Table 2). Capsule genotype diversity consisted of polysaccharides of types A and B, and lipopolysaccharides of types L2 and L3. The genotype diversity according to RIRDC MLST scheme included: ST122, ST460 and ST132. The genotype diversity based on Multi-host MLST scheme included ST64 and ST65. The genomes of isolates *LPG-3*, *LPG-5*, *LPG-9*, and *LPG-15* formed the genotype ^RIRDC^ST460, a derivative of genotype ^RIRDC^ST122, due to a silencing mutation in the ^RIRDC^pmi locus. However, isolates of both genotypes (^RIRDC^ST122 and ^RIRDC^ST460) remained identical according to the multi-host MLST (^mh^ST64) scheme.

Tested isolates contained all the genes encoding capsular polysaccharide and lipopolysaccharide biosynthesis (Supplementary Figure S1). Serogroup B isolates harbored a nonsense mutation at nucleotide position 282 of the *lpt-3* gene involved in the assembly of the inner core of LPS. Additionally, a deletion of the first half of the *lpt-3* gene, 864 bp in size (i.e., 54.44% of the reference gene), was detected in the *LPG-26* isolate (serogroup A).

### 3.4. Phylogenetic and Epidemiological Analysis

**Figure 1:**
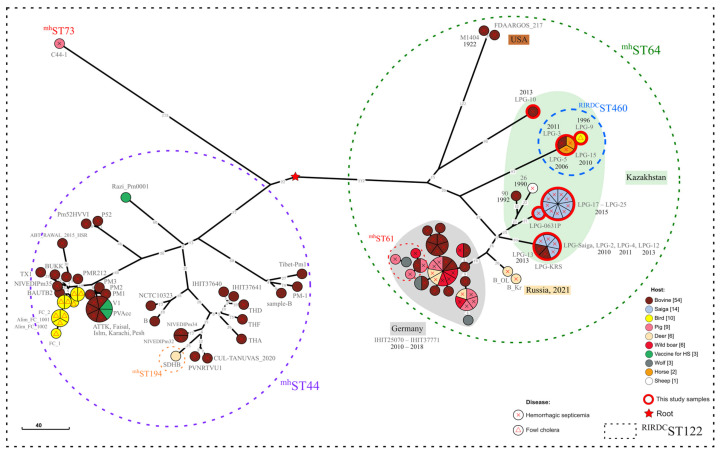
Maximum Parsimony phylogenetic tree based on *P. multocida* cgSNPs. Phylogenetic tree reconstructed based on 108 *P. multocida* genomes, including 21 isolates collected within current study. Overall, 1719 cgSNPs were identified, MP tree consists of 1772.

All serogroup B isolates were clustered in the B:L2:^RIRDC^ST122 clade, which predominantly consisted of strains with the ^mh^ST44 and ^mh^ST64 genotypes (Figure 1, Supplementary Table S2).

As shown in Figure 1, the ^mh^ST44 and ^mh^ST64 strains are separated by more than 400 cgSNPs. The ^mh^ST44 group of strains includes isolates from Pakistan (2008–2015), India (2000–2022), Thailand (2006–2011), Malaysia (2003), Bangladesh (2016–2021), Myanmar (1963), China (1995–2022), Iran (vaccine strain *Razi_Pm0001*, 1936), and Scotland (strain *1500C*, 2000). In addition to the isolates studied, the ^mh^ST64 strain group includes additional strains from Kazakhstan (strains *26* and *90*), as well as strains from Russia (*B_OL* and *B_Kr*), USA (*M1404* and *FDAARGOS_217*), and Germany (*IHIT25070*–*IHIT37771*).

Isolates recovered from saiga were grouped into two lineages associated with the Ural and Betpak-dala populations, separated by 70 cgSNPs. In the lineage associated with the Ural population, isolates *LPG-2*, *LPG-4*, *LPG-12*, *LPG-13*, *LPG-Saiga*, and *LPG-KRS* were identical by cgSNPs. However, only isolates *LPG-2* and *LPG-4* were associated with the mass mortality events of saiga in the Ural population in 2010 and 2011, respectively. Meanwhile, isolates *LPG-12* and *LPG-13* were collected in 2013 from saiga and cattle in the Karaganda region (Betpak-dala population) and East Kazakhstan region, respectively. In the branch associated with the Betpak-dala population, isolates *LPG-17* through *LPG-25* are identical by cgSNP. However, isolate *LPG-0631P* differs from them by one cgSNP, and is located in the earlier node within the MP tree, which is consistent with the earlier sample collection date.

Isolates *LPG-3*, *LPG-5*, *LPG-9*, and *LPG-15* belonged to ^RIRDC^ST460 genotype. Interestingly, these isolates were collected from different animal species over a wide range of dates (1996, 2006, 2010, and 2011) and distant geographic regions (West Kazakhstan, Kostanay Region, East Kazakhstan Region, Almaty Region). However, the maximum distance between them was only 2 cgSNPs, while the distance to phylogenetically related isolates was more than 173 cgSNPs.

The *LPG-10* isolate is almost equally distant from the branches associated with the Ural and Betpak-dala populations by 215 and 219 cgSNPs, respectively, and from the *M1404* and *FDAARGOS_217* strains (isolated in the USA from bison in 1922) by 213 cgSNPs, although it shares a common 15 cgSNPs long maternal branch with them. The closest strains to the isolates collected from saiga are not only those from Kazakhstan (strains *26* and *90*) and Russia (*B_OL* and *B_Kr*), but also strains from Germany, associated with HS outbreaks involving wolves as estimated vectors [53].

Isolate *LPG-26*, collected from a saiga that is not associated with mass mortality (selected in 2024 from Ural population), belongs to the genotype A:L3:^RIRDC^ST132 (^mh^ST65). According to the PubMLST database, isolates with identical genotype were strongly associated with pneumonia in cattle, sheep, and goats in the Netherlands, Germany, New Zealand, Australia, and China. These findings are consistent with the results of post-mortem observations showing that lesions in saiga carcass were restricted to the lungs, with no evidence of hemorrhagic septicemia signs in other organs.

### 3.5. Virulence Genes Analysis

**Figure 2:**
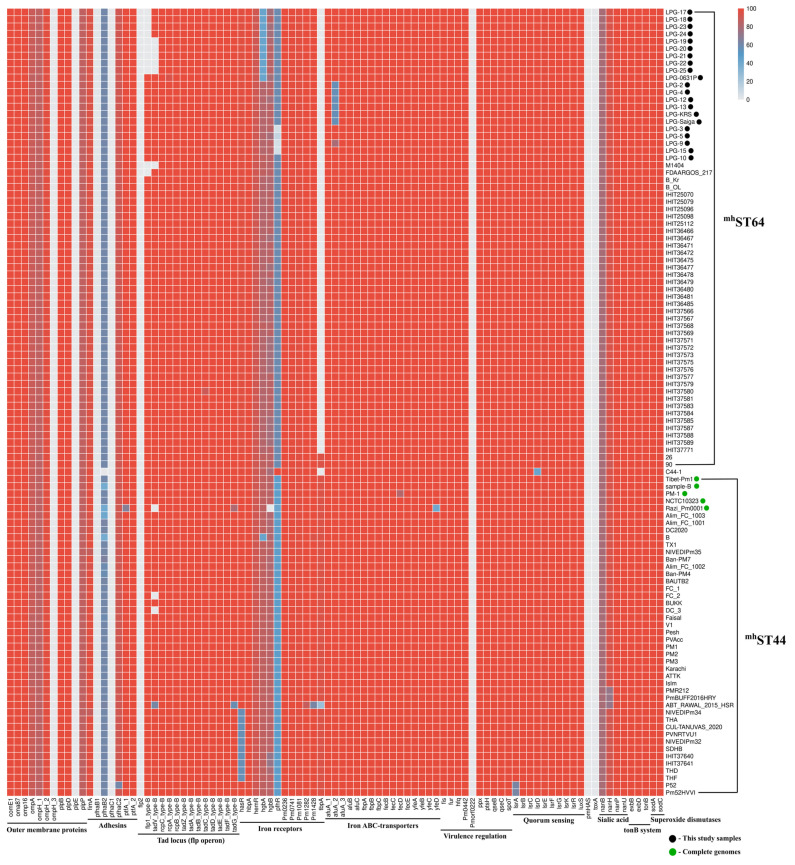
Heatmap representation of genotyping results for 91 genes encoding virulence factors in *P. multocida* isolates. Color scale represents percentages amino acid identity (%). All isolates are *P. multocida* draft genomes, with black dots indicating isolates collected from current study; green dots represent publicly available *P. multocida* complete genomes.

Genotyping of 21 isolates and 87 phylogenetically related serogroup B strains was performed for 91 genes encoding known virulence factors of *P. multocida* (Figure 2).

All isolates associated with the 2015 mass mortality event in the Betpak-dala population were found to have a deletion of the *flp1* gene, which encodes the main structural component of Flp pili—the *pre-Flp1* protein [54]. Isolates *LPG-19*, *LPG-20*, *LPG-21*, *LPG-22*, and *LPG-25* also had a deletion of the *tadV* gene, encoding prepilin peptidase, whose function is to proteolytically modify *pre-Flp1* into mature *Flp1*, as well as the pilin-like proteins *TadE* and *TadF* [55]. Deletions were confirmed by the absence of aligned raw reads on the reference *flp1* and *tadV* genes. Deletions of the *flp1* gene were also detected in strains *M1404* and *FDAARGOS_217*, while deletions of the *tadV* gene were also detected in strains *M1404*, *Razi_Pm0001*, *FC_2*, and *DC_3*. However, no such deletions were detected in strain *LPG-0631P*. Thus, the proportions of the *flp1* and *tadV* genes in the 108 genomes of the genotype B:L2:^RIRDC^ST122 are 89.81% and 91.66%, respectively. While the proportions of the presence of all other genes of the flp operon (*rcpC*, *rcpA*, *rcpB*, *tadZ*, *tadA*, *tadB*, *tadC*, *tadD*, *tadE*, *tadF* and *tadG*) are 100%.

Isolates *LPG-17*–*LPG-25*, as well as *LPG-0631P*, had an inactivated *hgbA* gene encoding hemoglobin-binding protein A, 927 amino acids (aa) in size (Supplementary Figure S2). Alignment with the sequences of this gene in other serogroup B strains revealed few differences, such as 630 bp deletion at the beginning of the gene (35.3% of the gene), and multiple single-nucleotide deletions and substitutions uniformly distributed throughout the gene. Interestingly, among the other strains of the ^mh^ST44 and ^mh^ST64 genotypes, the pathogenic strain *B* (*PmB*, GCF_002893985.1) studied earlier [56] had identical mutations in this gene, with the exception of several SNVs in common with the ^mh^ST44 strains.

Isolates belonging to the Ural branch (*LPG-2*, *LPG-4*, *LPG-12*, *LPG-13*, *LPG-Saiga*, and *LPG-KRS*) had a deletion that resulted in a nonsense mutation in the *afuA_2* gene, which encodes periplasmic iron (III)-binding protein. The deletion was confirmed by mapping the raw reads to the reference gene sequence. This mutation predicts the synthesis of a truncated protein of 193 aa (instead of 340 aa). In the other studied isolates, this gene is 100% identical to the reference sequence, with an exception of isolate *LPG-9*, which is also predicted to synthesize a truncated protein of 271 aa. These changes in the *afuA_2* gene were not observed in other strains of the ^mh^ST44 and ^mh^ST64 branches.

Isolates *LPG-3*, *LPG-5*, *LPG-9*, and *LPG-15*, genotype ^RIRDC^ST460, differ in deletions in the *pfhR* gene, which encodes the outer membrane heme-acquisition receptor. The deletions was confirmed by mapping the raw reads to the reference gene sequence. The reference sequence of the *pfhR* gene has an open reading frame (ORF) of 727 aa. In all strains of the ^mh^ST64 clade, the relative sequence of the *pfhR* gene contains two ORFs, 117 aa and 429 aa in size (Supplementary Figure S3A). These ORFs represent the beginning and end of the reference sequence, covering 75.1% of the reference gene. However, in isolate *LPG-5*, only the ORF encoding 117 aa is present. In contrast, isolates *LPG-3*, *LPG-9* and *LPG-15* contain only the 380 aa encoding ORF (a shortened 429 aa ORF).

Interestingly, in the ^mh^ST44 clade strains, the relative *pfhR* gene sequence contains two ORFs of 316 aa and 343 aa, which together cover 90.6% of the reference gene (Supplementary Figure S3B). The exception is the vaccine strain *Razi_Pm0001*, which contains two ORFs of 357 aa and 343 aa, which together cover 96.2% of the reference gene. The only comparable strain in which the *pfhR* gene is 100% identical to the reference sequence is the *C44-1* strain of genotype B:L2:^mh^ST73 (^RIRDC^ST122), isolated from a pig with HS.

Strains isolated from cattle in Thailand and India also had a distinctive feature. Strains *THA*, *THD*, *THF*, *SDHB*, *NIVEDIPm34*, *NIVEDIPm32*, *PVNRTVU1*, *CUL-TANUVAS_2020*, as well as *IHIT37640* and *IHIT37641*, had a deletion in the *hasR* gene, which encodes the heme acquisition system receptor. As a result of the deletion, which resulted in a nonsense mutation, the ORF size was 257 aa, compared to 848 aa in the reference gene. These strains are located on the same branch of the MP tree, explaining the inheritance of this trait.

Strains with the ^mh^ST64 genotype differed from ^mh^ST44 strains by the absence of the *tbpA* gene, which encodes transferrin binding protein A, however, with the exception of strains *26* and *90*, previously collected from Kazakhstan. Among all analyzed *P. multocida* serotype B:2 genomes genes the *toxA*, *Pmorf0222*, *pmHAS*, *flp2*, *ompH_3*, *pfhaB1*, *pfhaC1*, and *plpE* were not detected.

It is important to note that most of the single-gene deviations in virulence gene identity observed in ^mh^ST44 strains in Figure 2 were likely result of the low quality of the draft genome assemblies. For example, strains sequenced by the IonTorrent or 454 platforms, as well as those assembled by the Velvet assembler (*1500C*, *PMTB*, *HS_SKN01*, *VTCCBAA264*, *P52VAC*, *Alim_FC_1000*) were not used in this study. Therefore, strains with available SRA numbers (*ATTK*, *P52*, *SDHB*, and *BAUTB2*) were reassembled de novo using the SPAdes software with the “-k auto --carefull” parameters. The complete genome of the *Tibet-Pm1* strain (CP072655.1) corresponds to most of the strains in Figure 2, and was selected as the reference sequence of serogroup B in downstream analyses.

### 3.6. Tad Locus (flp Operon)

The tad locus was a particular interest in our analysis, as the *flp1* gene deletion was unique feature of isolates collected from the 2015 Betpak-dala outbreak. The flp operon of *P. multocida* has been discussed in previous research studies [20]; however, information on the structure and differences in tad locus for serogroups B and E was limited.

**Figure 3:**
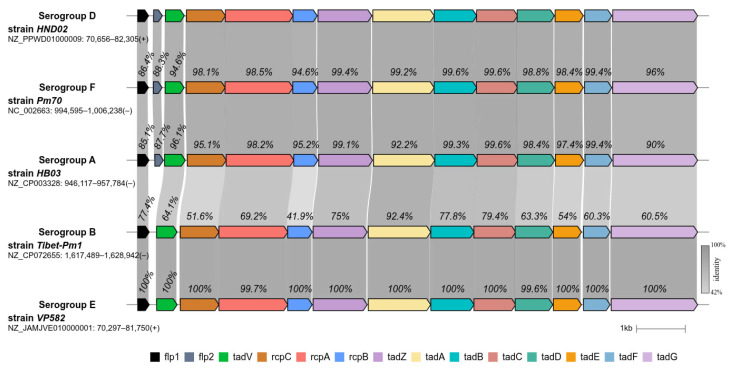
Visual representation of the main types of tad locus amino acid structure of *P. multocida* serogroups (A, B, D, E and F). Predominant tad locus architectures have shown for each serogroup, genes present shown in different colors, including percentages amino acid identities (%).

Amino acid alignment results show that the tad locus of serogroups causing HS are highly homologous both between serogroups and between strains (Figure 2). In comparison with serogroups A, F, and D, serogroups B and E lack the *flp2* gene and differ in their amino acid identity with other serogroups (Figure 3).

Figure 3 demonstrates the main types of tad locus across *P. multocida* serogroups, i.e., those found in the majority of isolates. In preliminary analyses, we observed different types of tad locus within serogroups. For example, serogroups A, F, and D may lack the *flp2* gene; strains belonging to genotype ^RIRDC^ST287 of serogroup D had a specific type of tad locus; and serogroup A strains exhibited a variety of different types with varying amino acid identities. Further analysis is needed to investigate specific patterns and variations of tad locus types across serogroups A, F, and D.

### 3.7. Mobile Genetic Elements

**Figure 4:**
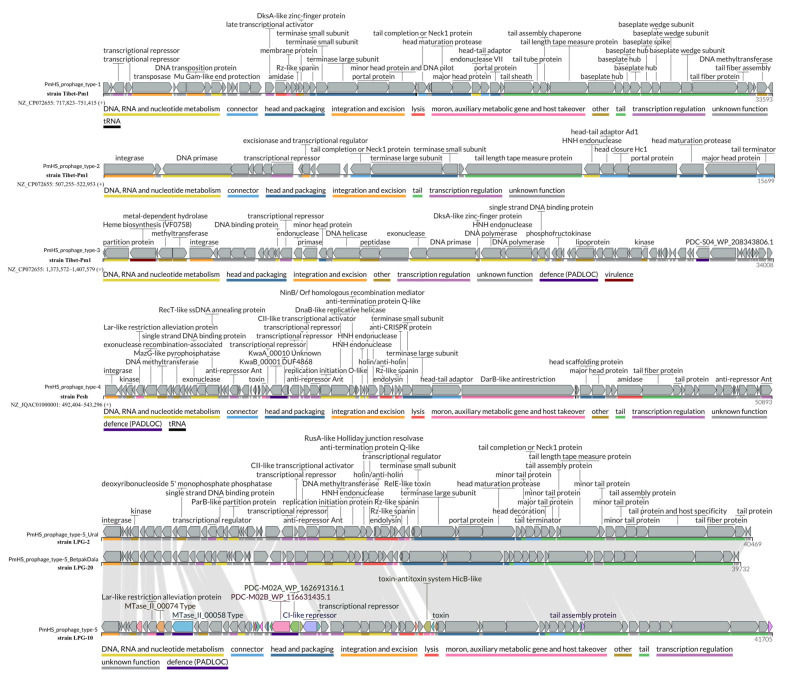
Annotation of the genomes of 5 types of *P. multocida* serogroup B prophages. Colors correspond to the genes’ assignment to specific protein functional annotation categories (shown under each type). The PmHS_prophage_type-5 genome annotation is represented by prophages from three isolates (*LPG-2*, *LPG-20* and *LPG-10*), with links indicating homologous genes.

Plasmid sequences, ICEs, AMR genes, or resistance-associated mutations in *P. multocida* genomes were not detected in the isolates studied. Analysis of the average nucleotide identity of prophage genomes from 108 genomes of the B:L2:^RIRDC^ST122 genotype identified five major groups of different prophage types, referred to in this study as “PmHS prophage types”. Prophages PmHS_prophage_type-1 and PmHS_prophage_type-2 were common across all strains (Supplementary Figure S4). Within the HS-associated isolates studied, with the exception of isolates of the ^RIRDC^ST460 genotype, previously unobserved 40.4 kbp prophage (PmHS_prophage_type-5) was detected. Isolates from Betpak-dala population were distinct from the isolates belonging to Ural population by a 199 aa deletion of the PmHS_prophage_type-5 prophage gene, encoding deoxyribonucleoside 5′ monophosphate phosphatase (UniProt: Q38167) (Figure 4). An evolutionarily altered homologue of the PmHS_prophage_type-5 prophage was identified in isolate *LPG-10*, covering 81% (with 95.2% nucleotide identity) of the analogous prophage genomes. Genome annotation of the PmHS_prophage_type-5 prophages did not reveal genes potentially involved in bacterial virulence or pathogenicity, similar to other types of *P. multocida* serogroup B prophages (annotated prophage genomes are available in Supplementary File S4).

### 3.8. Pangenome and Association Analyses

**Figure 5:**
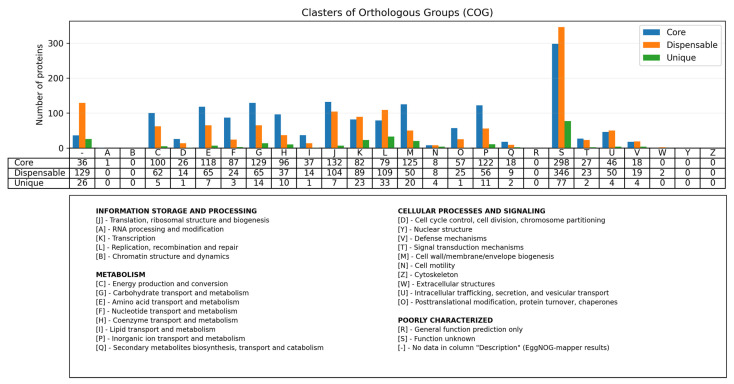
Functional annotation of representative clusters of orthologous genes of the *P. multocida* genotype B:L2:^RIRDC^ST122 pangenome. Core genes are shown in blue, dispensable genes are shown in orange, and unique genes are shown in green. One letter code descriptions correspond to the COG functional classification categories.

Analysis of 108 *P. multocida* genomes of the B:L2:^RIRDC^ST122 genotype revealed an open pangenome consisting of 1594 core genes (47.63%), 1421 dispansable genes (42.46%), and 331 unique genes (9.89%) (Supplementary Figure S5). Functional annotation of representative clusters of orthologous genes also confirms that core genes account for the majority of the pangenome (Figure 5). Association analysis, as well as annotation of unique genes of isolates associated with mass saiga mortality, revealed only genes of the PmHS_prophage_type-5 prophage.

## 4. Discussion

Based on the integrity of two genes encoding heptosyltransferases (*hptA* and *hptB*), all studied isolates express glycoforms A and B of the inner core LPS. Moreover, due to a nonsense mutation in the *lpt-3* gene, which is crucial for the addition of phosphoethanolamine (PEtn) to the 3rd position of the second heptose (Hep II), all studied serogroup B isolates belonged to lipopolysaccharide serovar 2, similar to strains from the USA, Pakistan, and Thailand [10].

The Multi-host MLST scheme demonstrated higher resolution, compared to the RIRDC MLST scheme, when analyzing strains associated with HS. However, the RIRDC MLST scheme is more informative when comparing strains associated with other pasteurellosis syndromes [20]. Overall, combining both genotyping schemes with the cgSNP-based phylogenetic clustering method provided a robust strain discrimination and extensive coverage of epidemiological data.

Our findings suggest that based on the phylogenetic position, differences in virulence genes, and the deleted PmHS_prophage_type-5 prophage gene, it can be concluded that outbreaks of HS in the Ural saiga population (2010–2011) and the outbreak in the Betpak-dala population (2015) were associated with genetically different strains of *P. multocida* genotype B:2:^RIRDC^ST122 (^mh^ST64). Notably, the genotype associated with mass mortality in the Ural population was observed several years later in saiga in the Betpak-dala population and in cattle on the opposite side of Kazakhstan (East Kazakhstan). This clearly demonstrates the transmission of the pathogen between different saiga populations, as well as between wild and livestock animals. Although the 2015 mass die-off was associated only with the Betpak-Dala saiga population, it is likely that the concurrent die-off of 10,358 saiga in the Ustyurt population was also related to this outbreak. However, we cannot confirm this hypothesis due to the limited isolates that were collected from this outbreak and their whole-genome data. Further research studies that focus on obtaining comprehensive genomic data from similar outbreaks will be crucial for deeper understanding of pathogen transmission.

Our results showed that genotyping based on virulence genes provided additional data for the epidemiological analysis of *P. multocida*. However, given the diversity of these genes due to mutations (especially deletions and nonsense mutations), it is recommended to analyze the complete ORF sequence. This may explain the variability of the results of PCR typing of virulence genes in many studies of *P. multocida*. Research study by Orynbayev M. et al. [23] investigated mass deaths of saiga in the Ural and Betpak-dala populations in 2010–2015 and, using similar *P. multocida* isolates, detected the *pmHAS* and *hgbA* genes by PCR typing. Whereas, our results and the results of Kutzer P. et al. [53] showed that *P. multocida* serotype B:L2 strains lack the *pmHAS* gene, and in isolates from the 2015 Betpak-dala outbreak, the *hgbA* gene was inactivated.

It is also important to note that for accurate genotyping of *P. multocida* virulence genes, PastyVRDB database may need to be updated [39] to account for different serogroups, as well as the *multocida* and *septica* subspecies. The fromAssembly2Feature software tool has no direct analogues, provides accurate results and is straightforward to use. However, in interim analyses, we observed that in rare cases the program did not determine the sequences of some genes. We assume that an atypical start codon or different sequences of the beginning of target genes in subject genome assemblies prevent the program from identifying ORFs, which can lead to a false negative result. Therefore, we suggest that the fromAssembly2Feature tool is used with caution and the results verified by additional tools. The development of a similar tool with higher accuracy or an improvement of the fromAssembly2Feature program remains a challenge for current bioinformatics analyses.

The tad locus genes encode the Flp pili assembly system required for colonization, nonspecific adhesion, pathogenesis, and biofilm formation in many members of the *Pasteurellaceae* family, including the *Actinobacillus*, *Haemophilus*, and *Pasteurella* genera [57]. Negative mutations in tad genes are known to result in a nonadherent avirulent phenotype of bacteria, suggesting the requirement for an intact flp operon [57,58]. For example, deletion of the *flp1-3* genes in *Haemophilus ducreyi* resulted in a significant reduction in the ability to cause disease in a human infection model [59]. While the mutant with a deletion of the *tadA* gene lost the ability to form microcolonies and pustules in humans. Insertion of a transposon into the *flp1* gene resulted in the loss of nonspecific adhesion in *Actinobacillus actinomycetemcomitans* [60]. Both *Actinobacillus pleuropneumoniae* mutants carrying deletions of the *flp1* or *tadD* genes lost cell surface pili, resulting in significant reductions in biofilm formation, cell adhesion, resistance to phagocytosis, survival in host whole blood, and colonization in vivo [61].

A comparative description of the tad locus of *P. multocida* serogroup B has not been previously performed. Our results show that *P. multocida* serogroups B and E are characterized by the presence of highly homologous 13 genes of the tad locus. To date, there is no experimental data confirming the involvement of the flp operon in the pathogenesis of HS, despite the fact that serogroup B strains are more suitable for a septicemic infection model in mice [62]. However, for *P. multocida* serogroup A (associated with bovine pneumonia), it was shown that transposon insertions into the *tadA* or *tadD* genes led to a significant decrease in pathogenicity in a septicemic infection model in mice [62]. The only study demonstrating the effect of lack of *flp1* gene expression was performed on a *P. multocida* serogroup A strain. In competitive growth assays, the mutant strain was stably attenuated in mice, but inconsistent results were observed in chickens [63].

In contrast to previous studies, archived *P. multocida* isolates *LPG-17* to *LPG-25* caused death of mice within 10 h after infection, despite the deletion of the *flp1* and *tadV* genes. Unfortunately, this does not provide a clear understanding whether flp operon dysfunction could contribute to the massive outbreak of pasteurellosis in the Betpak-dala population in 2015. Most likely, the primary mutation was the deletion of the *flp1* gene, since the deletion of the *tadV* gene is observed in only half of the isolates. We consider the following hypotheses for the origin of the *flp1* gene deletion: (A) the deletion could be a spontaneous event reinforced by positive selection facilitating the spread of infection; (B) the deletion could result from the reduction in the pathogen genome due to multiple replication in many infected animals; (C) and the deletion could be a consequence of bacterial growth in dead saiga carcasses due to the lack of active host immune defense pressure. Thus, an experimental assessment of virulence and pathogenicity in a septicemic mouse model using the *flp1* mutant strain of *P. multocida* serogroup B can be a promising direction for future research.

The absence of the *tbpA* gene in ^mh^ST64 strains, unlike ^mh^ST44 strains, was also determined in a study of German-Hungarian isolates [53]. The loss of the only transferrin-binding protein in *P. multocida* ^mh^ST64 was likely a consequence of spontaneous genome reduction in the common ancestor, since the *tbpA* gene was also present in other serogroups. Compensation for the loss of the transferrin iron source can be explained by the fact that a large proportion of iron ions in host animals is stored in heme, and the main source of heme absorption in bacteria is hemoglobin [64]. Under limited iron conditions, *P. multocida* expresses the hemoglobin-binding proteins *hgbA* and *hgbB* [65]. These heme receptors are physically identical to *tbpA* and directly recognize host hemoglobin. Quantitative in vitro and in vivo analyses showed that the virulence of the *P. multocida hgbA* mutant did not change, while the kinetics of hemoglobin binding slowed down [66]. Therefore, inactivation of the *hgbA* gene in isolates *LPG-17–LPG-25* and *LPG-0631P* was compensated by the *hgbB* gene without affecting its virulence.

Compensation for negative mutations in the iron acquisition genes in *P. multocida* occurred by the presence of these genes in multiple copies [64]. Unfortunately, *afuA_2* and *pfhR* genes were not well studied previously. It is known that the *afuA* gene is part of the afuCBA operon, which encodes an iron transport system similar to that found in *Actinobacillus pleuropneumoniae* [20]. However, due to the lack of data on the functional mechanism of this operon, we cannot say how a deletion in the *afuA_2* gene could affect the virulence of isolates collected from outbreaks in the Ural population. Given the conservation of several different forms of the *pfhR* gene (containing two ORFs) among strains ^mh^ST44 and ^mh^ST64, it appears that the entire sequence of the reference gene encodes functionally important three-dimensional structures. It is possible that the deletions of one of the ORF pairs of the *pfhR* gene observed in isolates *LPG-3*, *LPG-5*, *LPG-9* and *LPG-15* could be a consequence of adaptation to different host species.

Annotation of the PmHS_prophage_type-1, PmHS_prophage_type-2, and PmHS_prophage_type-5 genomes revealed no virulence or pathogenicity genes, similar to the pathogenesis of progressive atrophic rhinitis in porcine [67]. The deleted gene encoding deoxyribonucleoside 5′ monophosphate phosphatase (UniProt: Q38167) is likely responsible for the degradation of host cell deoxynucleoside 5′-monophosphates to deoxynucleosides and phosphate.

It has previously been published that the *P. multocida* species has an open pangenome [68]. Our results indicate that 108 *P. multocida* B:L2:^RIRDC^ST122 genomes also exhibit an open pangenome. The majority of the pangenome consists of core genes, taking into account that our dataset mostly reconstructed from draft genomes (95.37%), the proportion of core genes is actually underestimated.

One of the limitations of current research study is the lack of isolates collected from healthy saiga species in the studied populations. Comparison of isolates not associated with mass mortality would have allowed for a more reliable identification of significant genetic differences. It is also crucial to note that third-generation sequencing (TGS) of the isolates and subsequent assembly of complete genomes would have allowed for a more confident interpretation of identified or missing genetic features.

The first reported case of saiga death from pneumonia caused by the specific genotype A:L3:^RIRDC^ST132 (^mh^ST65) clearly demonstrates that saiga carry more than just the B:L2:^RIRDC^ST122 genotype. Therefore, saiga populations may be at risk not only from outbreaks of hemorrhagic septicemia but also from potential outbreaks of respiratory infection. Considering that the total saiga population in Kazakhstan reached a record 4.1 million individuals in July 2025, the epizootological service should expect new mass outbreaks of pasteurellosis. These outbreaks could significantly affect local population of wild and livestock species; therefore, proactive control measures and monitoring of the infection will be essential.

The results of our study demonstrate the epidemiology of historical cases of pasteurellosis in wild saigas and provide a basis for comparative analysis of future outbreaks. Comparing the genomic characteristics of the HS pathogen responsible for mass animal deaths with more recent isolates can help identify factors and transmission routes, particularly in regions where wildlife and livestock interact. Understanding these factors and routes is crucial for improving disease control on farms and preserving wild saiga populations.

## 5. Conclusions

In this study, we determined that mass deaths in the Ural (2010–2011) and Betpak-dala saiga populations (2015) were associated with different strains of *P. multocida* genotype B:2:^mh^ST64 (^RIRDC^ST122). Isolates collected from the outbreak in the Ural population had a large deletion in the *afuA_2* gene, while isolates from the outbreak in the Betpak-dala population had a deletion of the *flp1* gene. The impact of these mutations on the virulence of *P. multocida* serogroup B is unknown due to the lack of experimental data in the scientific literature. Plasmid sequences, ICEs, or antibiotic resistance genes were not detected in any of the studied isolates. However, a new prophage type was identified in saiga isolates, the annotation of which revealed no virulence or pathogenicity genes. Strains of the ^mh^ST64 genotype were found to lack transferrin binding protein A (*tbpA*). Analysis of 108 *P. multocida* genomes of the B:L2:^RIRDC^ST122 genotype revealed an open pangenome.

Although genetic features that clearly describe the high virulence of *P. multocida* isolates associated with mass saiga mortality were not detected, this study represents the first comprehensive whole genome and comparative genomic analysis of *P. multocida* from saiga mass death events.

## Figures and Tables

**Table 1:** Summary of metadata for the *Pasteurella multocida* isolates analyzed in current study.

Isolate	Host Species	Disease	Sampling Date	Region	Coordinates	Saiga Population
LPG-2	saiga	HS	1 May 2010	West-Kazakhstan Region	48°33′ N 49°34′ E	Ural
LPG-3	cattle	HS	1 April 2011	West-Kazakhstan Region	50°34′ N 49°40′ E	
LPG-4	saiga	HS	1 June 2011	West-Kazakhstan Region	49°27′ N 46°53′ E	Ural
LPG-5	horse	HS	23 December 2006	Kostanai Region	53°25′ N 64°18′ E	
LPG-9	poultry	HS	24 March 1996	East-Kazakhstan Region	50°24′ N 80°13′ E	
LPG-10	cattle	FC	16 October 2013	East-Kazakhstan Region	48°46′ N 84°42′ E	
LPG-12	saiga	HS	2 September 2013	Karaganda Region	49°52′ N 68°51′ E	Betpak-dala
LPG-13	cattle	HS	2 September 2013	East-Kazakhstan Region	48°34′ N 83°39′ E	
LPG-15	horse	HS	14 January 2010	Almaty Region	43°11′ N 76°37′ E	
LPG-17	saiga	HS	20 May 2015	Aktobe Region	48°37′ N 61°16′ E	Betpak-dala
LPG-18	saiga	HS	20 May 2015	Aktobe Region	48°37′ N 61°16′ E	Betpak-dala
LPG-19	saiga	HS	15 May 2015	Kostanai Region	49°36′ N 65°10′45″ E	Betpak-dala
LPG-20	saiga	HS	15 May 2015	Kostanai Region	49°36′ N 65°10′45″ E	Betpak-dala
LPG-21	saiga	HS	15 May 2015	Kostanai Region	49°36′ N 65°10′45″ E	Betpak-dala
LPG-22	saiga	HS	15 May 2015	Kostanai Region	49°36′ N 65°10′45″ E	Betpak-dala
LPG-23	saiga	HS	15 May 2015	Kostanai Region	50°11′05″ N 65°11′13″ E	Betpak-dala
LPG-24	saiga	HS	15 May 2015	Kostanai Region	50°11′05″ N 65°11′13″ E	Betpak-dala
LPG-25	saiga	HS	26 May 2015	Akmola Region	51°54′ N 67°19′ E	Betpak-dala
LPG-26	saiga	HS	2024	Atyrau Region	47°50′ N 47°54′ E	Ural
LPG-KRS	cattle	Ø	Ø	Ø	Ø	
LPG-Saiga	saiga	HS	Ø	Ø	Ø	Ø
LPG-0631P	saiga	HS	2012	Ø	Ø	Ø

Notes: Ø—no data available, HS—hemorrhagic septicemia, FC—fowl cholera.

**Table 2:** Summary of the genome assembly quality metrics of the studied *P. multocida* isolates.

Isolate	Number of Contigs *	*N50* *	Genome Size, Kbp *	Sequencing Depth, ×	Completeness/ Contamination, %	Capsule: LPS	RIRDC MLST	Multi-Host MLST
*LPG-2*	23	382993	2.30	56	99.55/0.00	B:L2	ST122	ST64
*LPG-3*	25	238125	2.26	59	99.55/0.00	B:L2	ST460	ST64
*LPG-4*	21	382993	2.30	58	99.55/0.00	B:L2	ST122	ST64
*LPG-5*	26	145857	2.25	57	99.55/0.00	B:L2	ST460	ST64
*LPG-9*	24	164157	2.26	57	99.55/0.00	B:L2	ST460	ST64
*LPG-10*	26	383218	2.30	65	99.55/0.00	B:L2	ST122	ST64
*LPG-12*	33	121393	2.29	56	99.55/0.00	B:L2	ST122	ST64
*LPG-13*	20	382993	2.30	76	99.55/0.00	B:L2	ST122	ST64
*LPG-15*	20	537706	2.26	45	99.55/0.00	B:L2	ST460	ST64
*LPG-17*	30	203092	2.29	58	99.55/0.00	B:L2	ST122	ST64
*LPG-18*	79	74370	2.30	64	99.55/0.00	B:L2	ST122	ST64
*LPG-19*	40	268459	2.30	65	99.55/0.00	B:L2	ST122	ST64
*LPG-20*	30	289533	2.30	59	99.55/0.00	B:L2	ST122	ST64
*LPG-21*	22	343344	2.30	64	99.55/0.00	B:L2	ST122	ST64
*LPG-22*	22	382993	2.30	52	99.55/0.00	B:L2	ST122	ST64
*LPG-23*	24	382737	2.29	92	99.55/0.00	B:L2	ST122	ST64
*LPG-24*	24	382737	2.29	66	99.55/0.00	B:L2	ST122	ST64
*LPG-25*	24	289686	2.30	63	99.55/0.00	B:L2	ST122	ST64
*LPG-26*	94	71346	2.29	97	98.41/0.00	A:L3	ST132	ST65
*LPG-KRS*	24	382993	2.30	56	99.55/0.00	B:L2	ST122	ST64
*LPG-Saiga*	21	382993	2.30	83	99.55/0.00	B:L2	ST122	ST64
*LPG-0631P*	82	64640	2.28	40	99.55/0.11	B:L2	ST122	ST64

Notes: *—statistics are based on analysis of contigs ≥ 500 bp.

## Data Availability

This whole-genome shotgun project has been deposited in BioProject under accession number PRJNA556768. The data from this study may be provided by the corresponding author upon reasonable request.

## Supplementary Materials

The following supporting information can be downloaded at: https://www.mdpi.com/article/10.3390/pathogens14121273/s1, Figure S1: Schematic representation of the genes encoding capsular polysaccharide and lipopolysaccharide biosynthesis in all *Pasteurella multocida* isolates, showing amino acid percentage identity and nonsense mutations and deletions in genes; Figure S2: A. Translation of the coding sequence of the hgbA gene encoding hemoglobin-binding protein A; B. Translation of the *hgbA* gene of *LPG-17*–*LPG-25*, as well as *LPG-0631P Pasteurella multocida* isolates; Figure S3: A. Open reading frame (*pfhR* gene) of all strains of the ^mh^ST64 clade; B. Open reading frame (*pfhR* gene) of all strains of the ^mh^ST44 clade; C. Open reading frame (pfhR gene) of Razi_Pm0001 strain; Figure S4: Heatmap representing the average nucleotide identity of prophage genomes, from 108 genomes of the B:L2:^RIRDC^ST122 genotype with identified five major groups of different prophage types; Figure S5: (A,B): Analysis of *P. multocida* pangenome of the B:L2:^RIRDC^ST122 genotype consisting of 1594 core genes (47.63%), 1421 dispansable genes (42.46%), and 331 unique genes (9.89%). A. Plot representing the number of genomes versus number of clusters; B. Plot representing number of genomes and corresponding number of genes; Table S1: Summary of parameters of the software tools used for the analysis of whole genome sequencing data in this study; Table S2: Summary metadata of the 108 *Pasteurella multocida* serogroup B isolates represented in maximum parsimony phylogenetic tree based on cgSNPs (Figure 1); File S1: Nucleotide sequences corresponding to *Pasteurella multocida* capsule and LPS biosynthesis genes, as well as virulence and antibiotic resistance genes that were detected using fromAssembly2Feature software based on PastyVRDB database; File S2: Script code to generate consensus alignments of raw reads to reference sequences of genes missing from the *P. multocida* genome assemblies; File S3: Script code for pangenome analysis used with Panaroo output data (“gene_presence_absence_filt_pseudo_length_frag”); File S4: Genome annotation of the *P. multocida* serotype B:L2 prophages.

## Abbreviations

The following abbreviations are used in this manuscript:

HS	Hemorrhagic septicemia
MLST	Multi-Locus Sequence Typing
LPS	Lipopolysaccharide
cgSNP	Core genome single nucleotide polymorphism
