# Supplementary material for: Genomic Insights into Pasteurella multocida Serotype B:2 from Hemorrhagic Septicemia Outbreaks in Wildlife and Livestock in Kazakhstan
Source: Pathogens. 2025 Dec 11;14(12):1273. doi: 10.3390/pathogens14121273 (PMC12735514; doi:10.3390/pathogens14121273)
Supplement: Supplementary file 1 [file pathogens-14-01273-s001.zip › pathogens-4006838-supplementary/Ethics_scan.pdf]

13/5, Kurgalzhynskoye road, Astana, 010000, Kazakhstan
Tel.: +7 (7172) 70-75-65
E-mail: info@biocenter.kz

EXTRACT

from the meeting No. 6 of the local ethics commission (hereinafter LEC) of the LLP "National center for biotechnology"

AGENDAS:

Discussion of the study protocol "Whole genome sequencing and analysis of *P. multocida* genomes associated with cases of hemorrhagic septicemia in saigas and livestock in Kazakhstan".

DISCUSSION:

Dr. Kanatbek Mukantayev, Chairman of the Local Ethics Committee of the National Center for Biotechnology, presented a study protocol aimed at "determining the distinctive genetic characteristics of *P. multocida* isolates associated with the mass mortality of saiga antelopes and livestock in Kazakhstan". The collection of pathological material and subsequent infection of laboratory mice for the purpose of assessing the pathogenicity of *Pasteurella multocida* will be carried out by trained veterinarians at the National Reference Center for Veterinary Medicine in accordance with the National Standard of the Republic of Kazakhstan ST RK 3508 "Methods of laboratory diagnostics of pasteurellosis".

CONCLUSION:

Considering that:

1) the collection of pathological material will be carried out subject to permission (from government agencies or the owners of the affected animals) and the signing of the corresponding questionnaire;

2) the infection of laboratory mice will be carried out according to state standards, adhering to international bioethical norms for experiments on laboratory animals;

The local ethics committee has decided to approve the study protocol "Whole genome sequencing and analysis of *P. multocida* genomes associated with cases of hemorrhagic septicemia in saigas and livestock in Kazakhstan."

Chairman of the Ethics Committee **K. Mukantayev**

Secretary of the Ethics Commission **M. Rakhimbergenova**

Astana city October 5, 2022

Present: members of the LEC

Data: ______________

INFORMED CONSENT FORM FOR ANIMAL OWNER - USE OF ANIMALS IN RESEARCH

Title of Study: Whole genome sequencing and analysis of *P. multocida* genomes associated with cases of HS in saigas and livestock in Kazakhstan.
Principal Investigator (PI): Asylulan Amirgazin
Contact details for this study: asylulan0894@gmail.com, +77023847557

We would like to invite you to participate in a research study. This form is to provide information to help you decide whether or not you want your animal to participate in the study. Please read this form carefully. You are encouraged to ask any questions about this study before deciding to participate.

PURPOSE OF THIS RESEARCH STUDY:
The purpose of the study is determine the distinctive genetic characteristics of *P. multocida* isolates associated with mass mortality of saiga antelopes and livestock in Kazakhstan.

WHAT IS INVOLVED IN PARTICIPATION IN THIS RESEARCH STUDY:
The study will involve collecting pathological material from animals (saigas, livestock, and others) that died from hemorrhagic septicemia due to pasteurellosis infection. The pathological material will be used to infect white laboratory mice to test the pathogenicity of the *P. multocida* isolates. Genomic DNA will be isolated from pure pathogen cultures, and whole genome sequencing will be performed.

VOLUNTARY PARTICIPATION AND RIGHT TO WITHDRAW

You don't have to be part of this research study if you don't want to, participation is voluntary. If you decide not to be in it, it won't cause any problems. You can take your animal out of the study whenever you want, and you won't be in trouble or have to explain why. If you choose not to be in the study or if you take your animal out, it won't change how well your animal is taken care of. The person in charge can also decide to take your animal out of the study if needed. If your animal is taken out of the study for any reason, the information already collected may still be used for research or learning purposes.

CONFIDENTIALITY

The information you share will be private. Only your name and your animal's name will be on the consent form, and it will be kept in a secure, lockable place. The things learned from this research might be shared for learning or regulatory reasons. If you want to know about the study's results, you can ask the person in charge, the principal investigator. They'll make sure to keep you and your animal's identity private.

POSSIBLE DISCOMFORTS AND RISKS:
To confirm the pathogenicity of the isolates, 0.2 ml of the corresponding 10% suspension will be administered subcutaneously to white laboratory mice, in accordance with the National Standard of the Republic of Kazakhstan ST RK 3508 "Methods of Laboratory Diagnostics of Pasteurellosis." As a result of infection, mice may develop an infection that can lead to severe illness or death. If the mice die, their cardiac blood will be used for culture on meat-peptone agar (HiMedia, India) and meat-peptone broth (HiMedia, India) for 24 hours at 37°C.

Data: ______________

INFORMED CONSENT FORM FOR ANIMAL OWNER - USE OF ANIMALS IN RESEARCH

POSSIBLE BENEFITS OF THE STUDY:

The results of the study will help diagnose pasteurellosis infection in dead animals, characterize the level of pathogenicity, and obtain whole genome sequences of *P. multocida* isolates.

UNFORESEEN RISKS

Sometimes, unexpected problems may happen during the research study. The research study investigators and people in charge will communicate to you as fast as possible of any new information that might influence your decision to allow your animal to continue in the study.

FINANCIAL IMPLICATIONS AND COMPENSATION

Having your animal participate in this research study won't cost you anything. If there are procedures done just for the study, you won't have to pay for them. You won't get any money for having your animal in the study. If there are other costs unrelated to the study like for diagnosing, managing, or treating your animal, those are your responsibility.

QUESTIONS ABOUT THIS STUDY

Research study investigators

If you have any questions or concerns about the study, possible discomforts, risks and benefits, your obligations or any other questions please feel free to contact the research personnel at any point.

Institutional Animal Care and Use Committee

This research study has been approved by the National Standard of the Republic of Kazakhstan ST RK 3508 "Methods of laboratory diagnostics of pasteurellosis".

Data: ______________

INFORMED CONSENT FORM FOR ANIMAL OWNER - USE OF ANIMALS IN RESEARCH

Owner's Consent for animals study participation

This research study has been explained to me and I give my consent that my animal can take part in the study. I have had the opportunity to ask questions and I understand that I can contact the research investigator via email or the phone number listed above, if I have additional questions or concerns.

If biological material is collected such as tissues or fluids, I give additional consent for the material to be stored and used for any future research/educational purposes (please tick box):

N/A: ☐ I give consent: ☐ I do not give consent: ☐

I certify with my signature that I am the legal owner or custodian of the animal and have the authority to consent to the animal participating in this research study.

Name and identification of Animal:

Name:

Species:

Breed:

Sex:

Age:

Name of Owner:

Name ______________________ Signature ______________________ Date __________

Research Study Investigator:

Name ______________________ Signature ______________________ Date __________

Acrobat Reader (available free here) is recommended for completion.

The ARRIVE guidelines 2.0: author checklist

The ARRIVE Essential 10

These items are the basic minimum to include in a manuscript. Without this information, readers and reviewers cannot assess the reliability of the findings.

Item		Recommendation	Section/line number, or reason for not reporting
Study design	1	For each experiment, provide brief details of study design including:<br>a. The groups being compared, including control groups. If no control group has been used, the rationale should be stated.<br>b. The experimental unit (e.g. a single animal, litter, or cage of animals).	Described in section 2.2
Sample size	2	a. Specify the exact number of experimental units allocated to each group, and the total number in each experiment. Also indicate the total number of animals used.<br>b. Explain how the sample size was decided. Provide details of any *a priori* sample size calculation, if done.	Described in section 2.2
Inclusion and exclusion criteria	3	a. Describe any criteria used for including and excluding animals (or experimental units) during the experiment, and data points during the analysis. Specify if these criteria were established *a priori*. If no criteria were set, state this explicitly.<br>b. For each experimental group, report any animals, experimental units or data points not included in the analysis and explain why. If there were no exclusions, state so.<br>c. For each analysis, report the exact value of *n* in each experimental group.	Described in section 2.2
Randomisation	4	a. State whether randomisation was used to allocate experimental units to control and treatment groups. If done, provide the method used to generate the randomisation sequence.<br>b. Describe the strategy used to minimise potential confounders such as the order of treatments and measurements, or animal/cage location. If confounders were not controlled, state this explicitly.	Described in section 2.2
Blinding	5	Describe who was aware of the group allocation at the different stages of the experiment (during the allocation, the conduct of the experiment, the outcome assessment, and the data analysis).	Described in section 2.2
Outcome measures	6	a. Clearly define all outcome measures assessed (e.g. cell death, molecular markers, or behavioural changes).<br>b. For hypothesis-testing studies, specify the primary outcome measure, i.e. the outcome measure that was used to determine the sample size.	Described in sections 2.2 and 3.1
Statistical methods	7	a. Provide details of the statistical methods used for each analysis, including software used.<br>b. Describe any methods used to assess whether the data met the assumptions of the statistical approach, and what was done if the assumptions were not met.	Described in section 2.2
Experimental animals	8	a. Provide species-appropriate details of the animals used, including species, strain and substrain, sex, age or developmental stage, and, if relevant, weight.<br>b. Provide further relevant information on the provenance of animals, health/immune status, genetic modification status, genotype, and any previous procedures.	Described in section 2.2
Experimental procedures	9	For each experimental group, including controls, describe the procedures in enough detail to allow others to replicate them, including:<br>a. What was done, how it was done and what was used.<br>b. When and how often.<br>c. Where (including detail of any acclimatisation periods).<br>d. Why (provide rationale for procedures).	Described in section 2.2
Results	10	For each experiment conducted, including independent replications, report:<br>a. Summary/descriptive statistics for each experimental group, with a measure of variability where applicable (e.g. mean and SD, or median and range).<br>b. If applicable, the effect size with a confidence interval.	Described in section 3.1
