# Supplementary figures and images for: Genomic Insights into Pasteurella multocida Serotype B:2 from Hemorrhagic Septicemia Outbreaks in Wildlife and Livestock in Kazakhstan
Source: Pathogens. 2025 Dec 11;14(12):1273. doi: 10.3390/pathogens14121273 (PMC12735514; doi:10.3390/pathogens14121273)

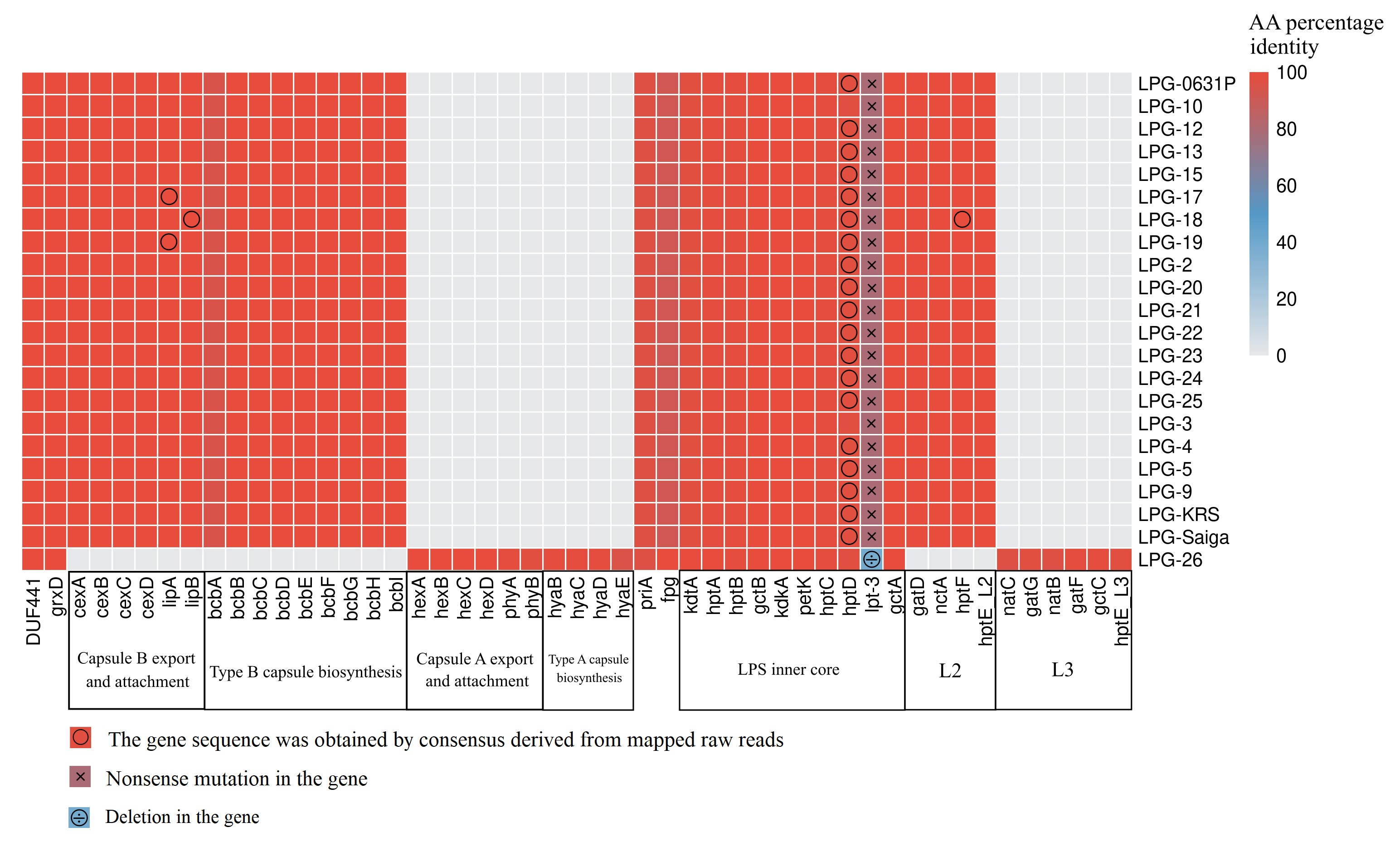

Supplement: Supplementary file 1 [file pathogens-14-01273-s001.zip › pathogens-4006838-supplementary/Supplementary Figures/Figure S1.jpg]

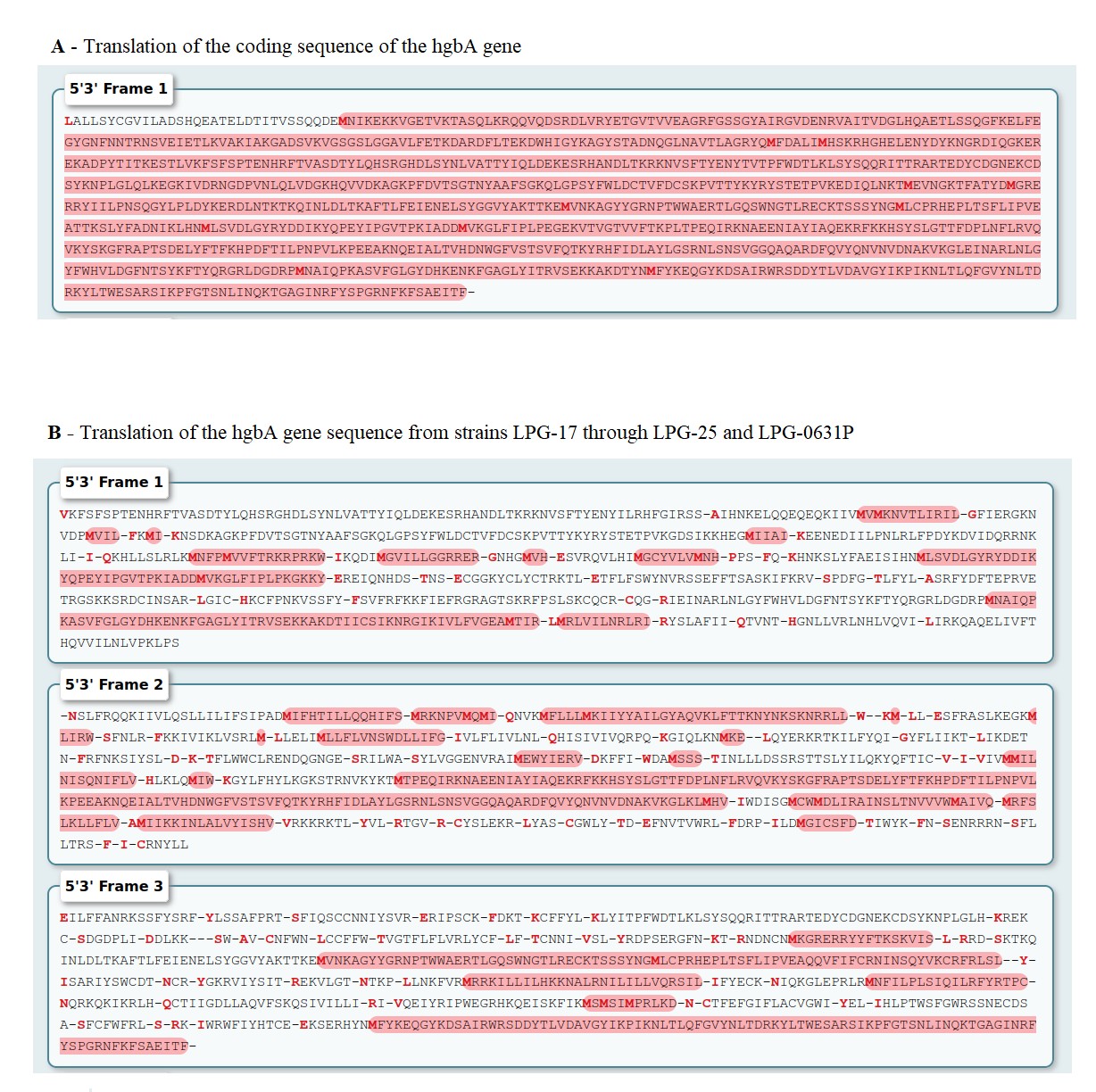

Supplement: Supplementary file 1 [file pathogens-14-01273-s001.zip › pathogens-4006838-supplementary/Supplementary Figures/Figure S2.jpg]

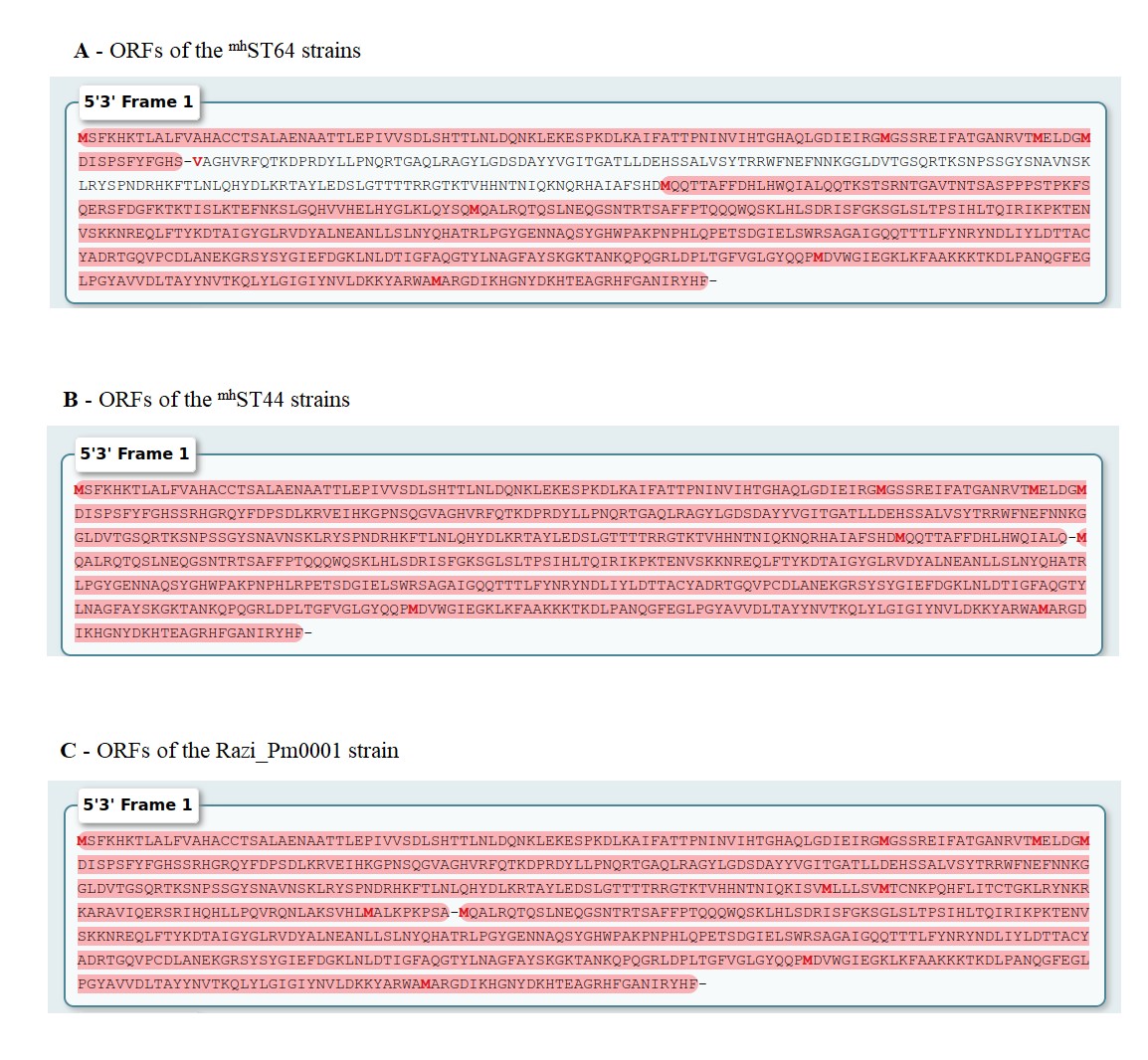

Supplement: Supplementary file 1 [file pathogens-14-01273-s001.zip › pathogens-4006838-supplementary/Supplementary Figures/Figure S3.jpg]

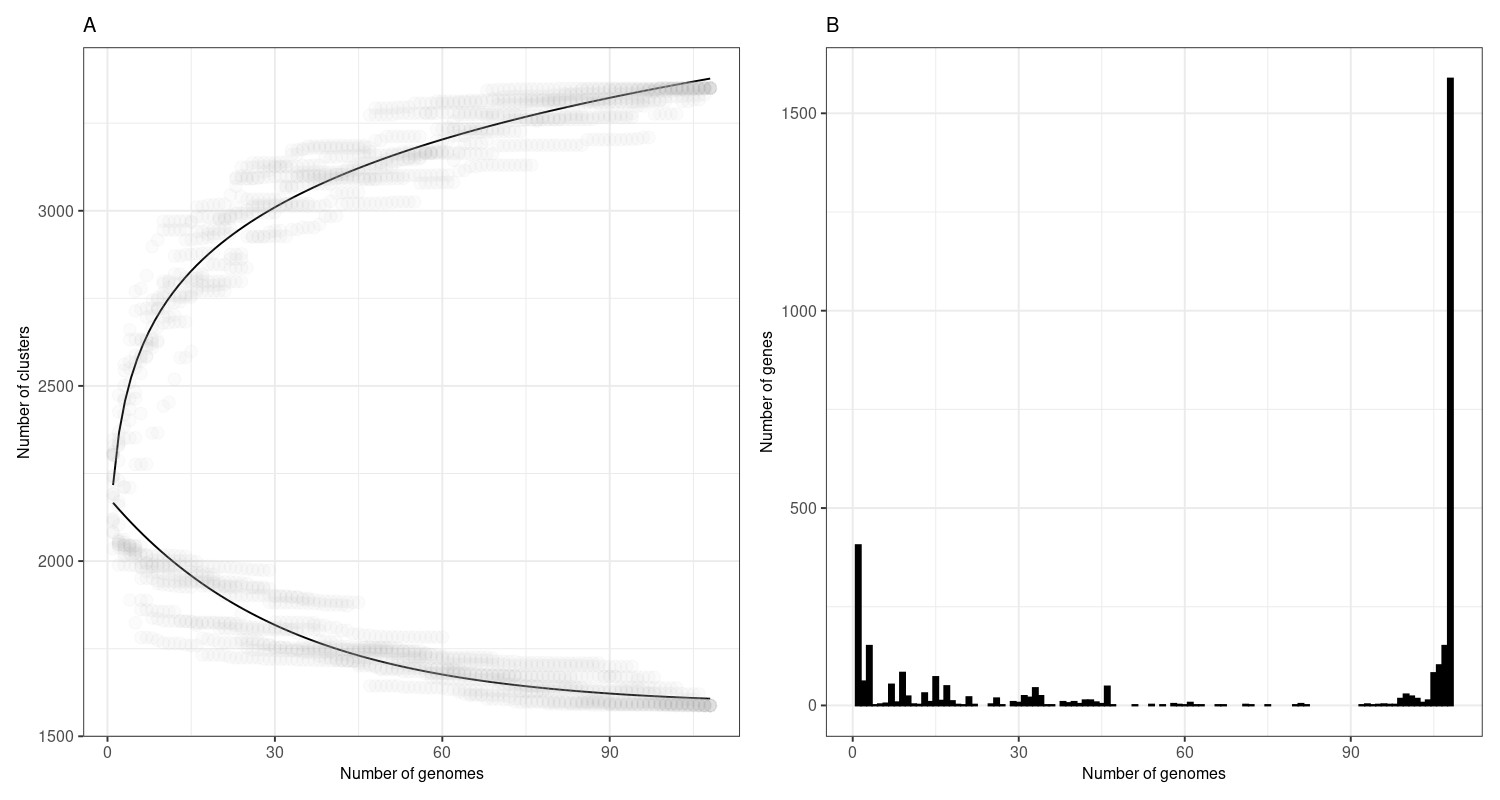

Supplement: Supplementary file 1 [file pathogens-14-01273-s001.zip › pathogens-4006838-supplementary/Supplementary Figures/Figure S5.jpg]
